# Supplementary figures and images for: T1SEstacker: A Tri-Layer Stacking Model Effectively Predicts Bacterial Type 1 Secreted Proteins Based on C-Terminal Non-repeats-in-Toxin-Motif Sequence Features
Source: Front Microbiol. 2022 Feb 8;12:813094. doi: 10.3389/fmicb.2021.813094 (PMC8861453; doi:10.3389/fmicb.2021.813094)

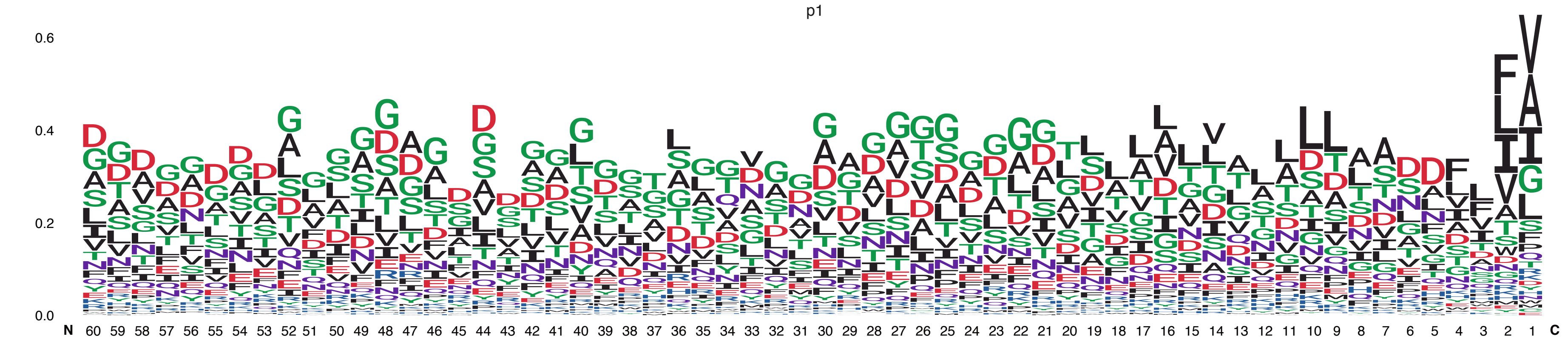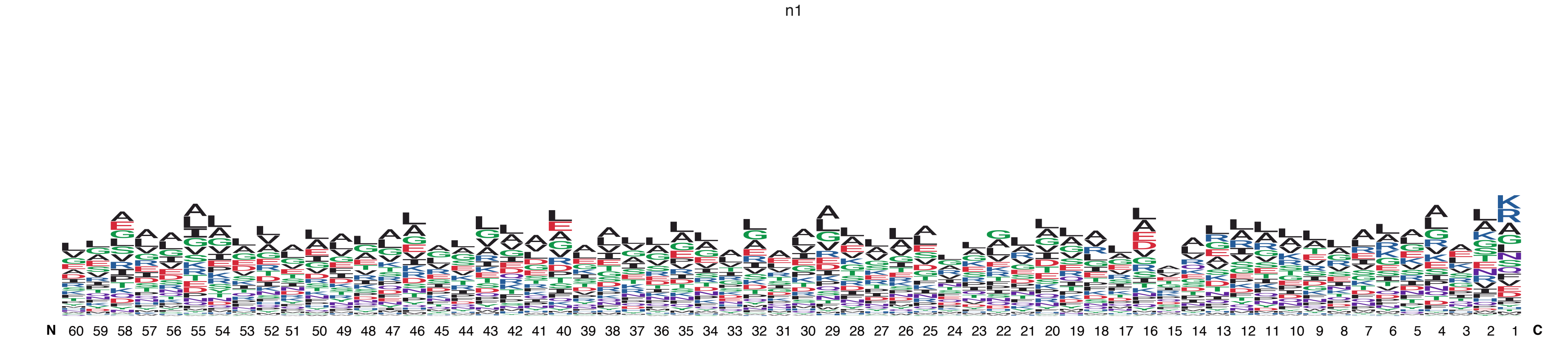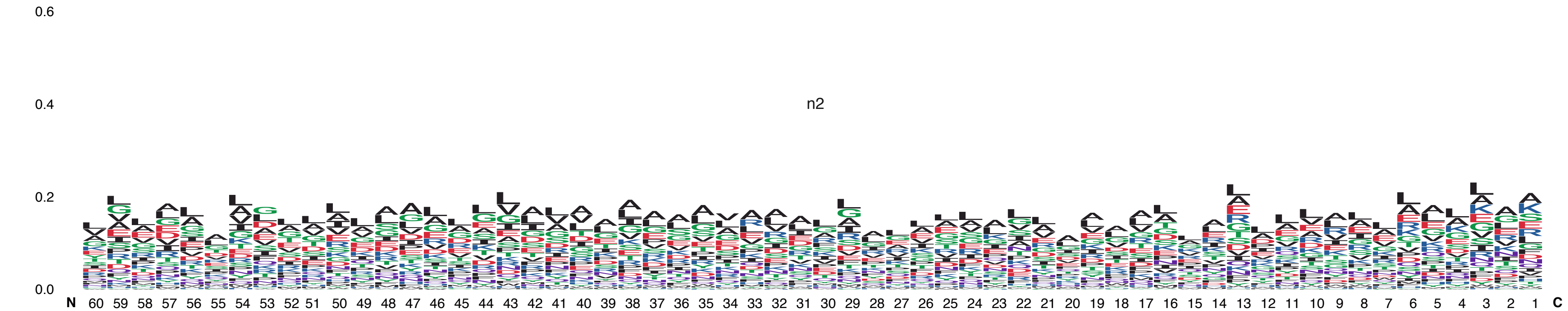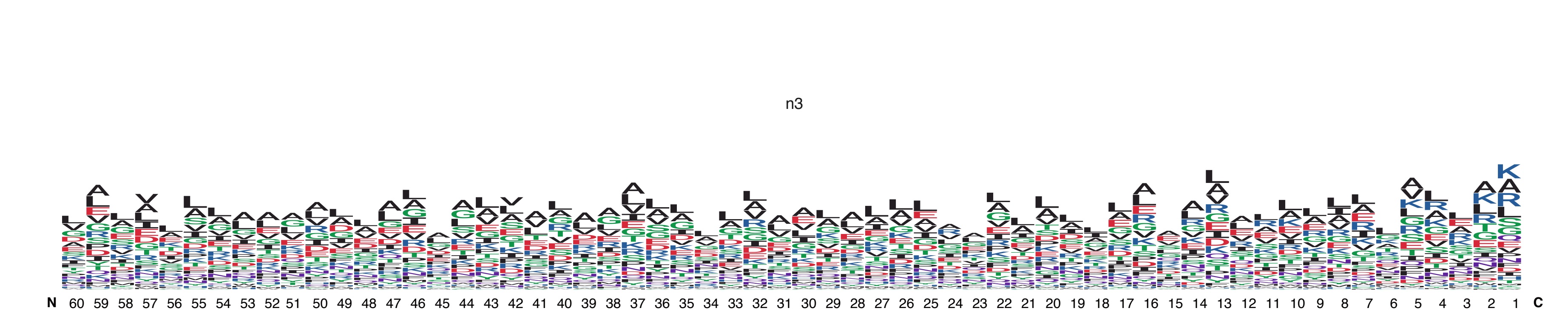

chemistry

|        |       |             |         |       |
|--------|-------|-------------|---------|-------|
| Acidic | Basic | Hydrophobic | Neutral | Polar |
|--------|-------|-------------|---------|-------|

Supplement: Supplementary Figure 1 — Position-specific Aac profile difference between the C termini of repeats-in-toxin (RTX) proteins and three independent groups of non-RTX proteins. [file Image_1.pdf]

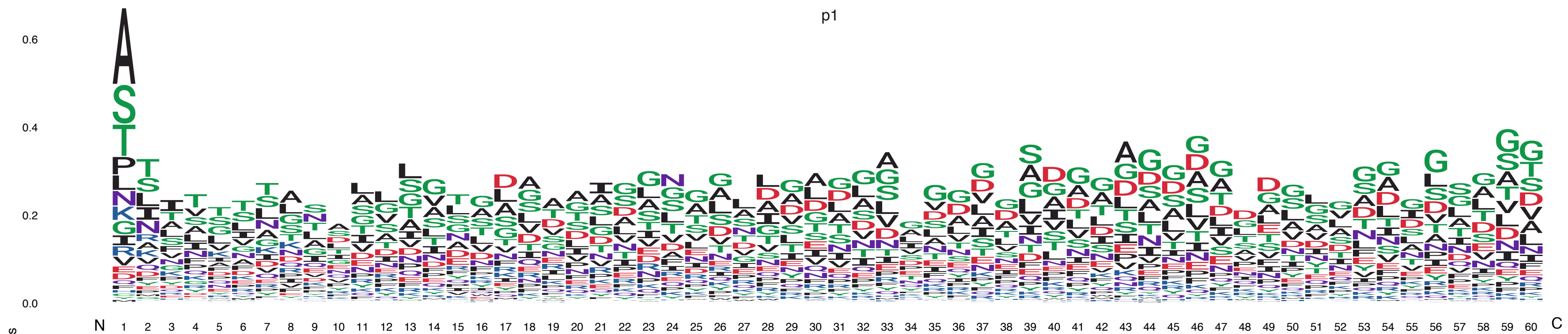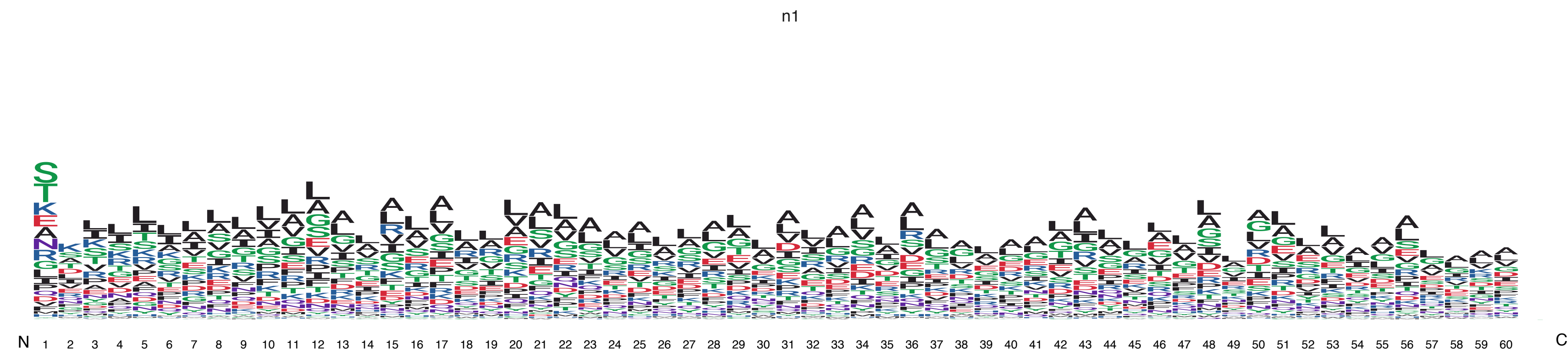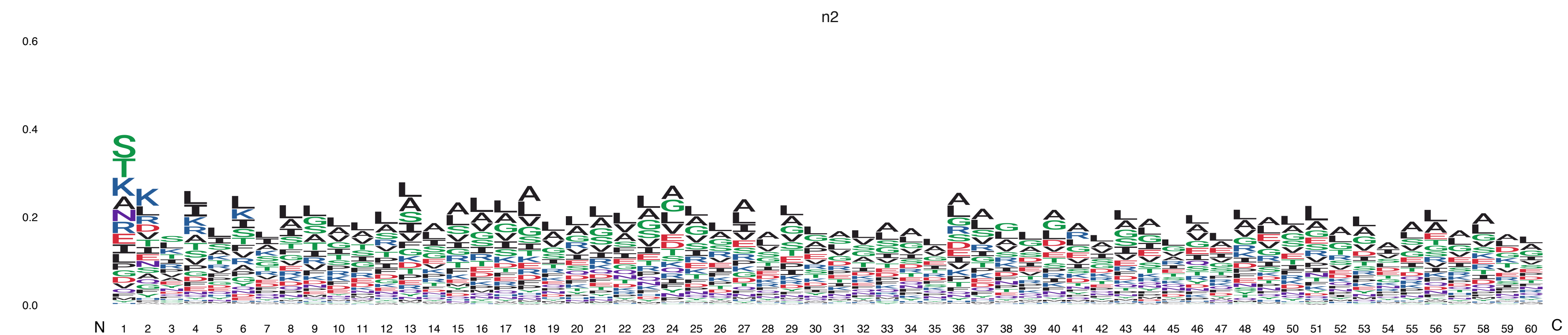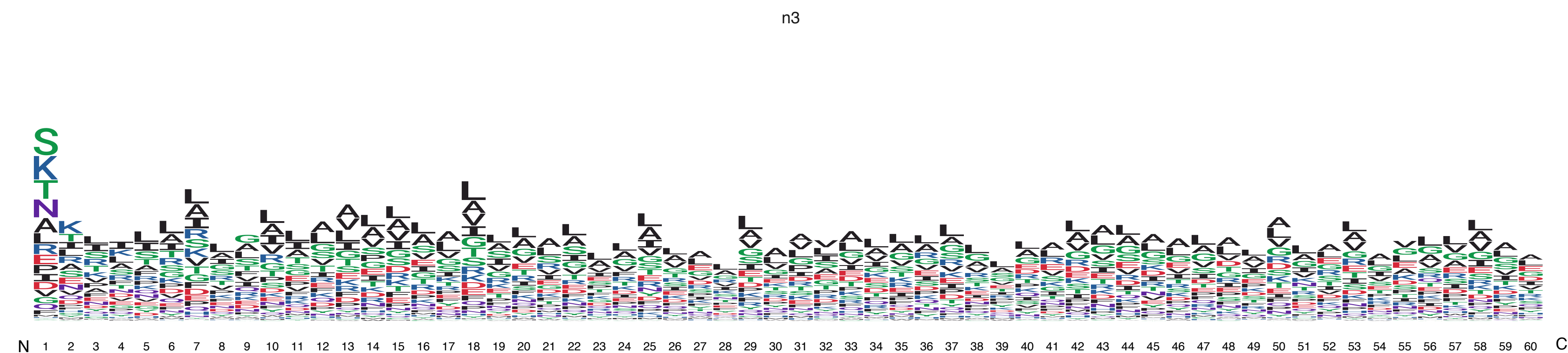

chemistry

|        |       |             |         |       |
|--------|-------|-------------|---------|-------|
| Acidic | Basic | Hydrophobic | Neutral | Polar |
|--------|-------|-------------|---------|-------|

Supplement: Supplementary Figure 2 — Position-specific Aac profile difference between the N termini of RTX proteins and three independent groups of non-RTX proteins. [file Image_2.pdf]

**A**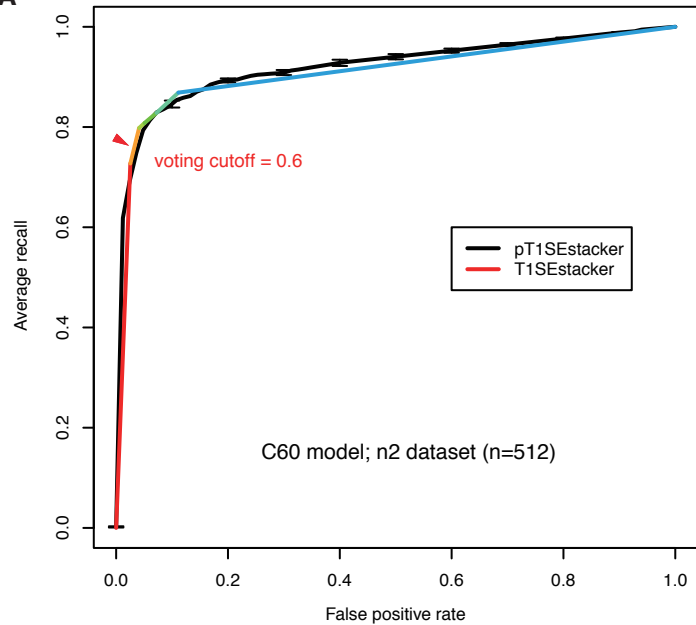**B**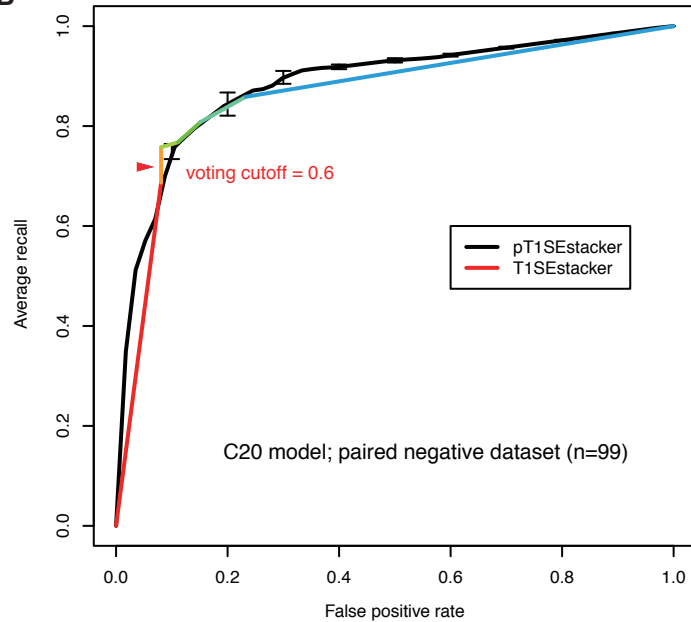**C**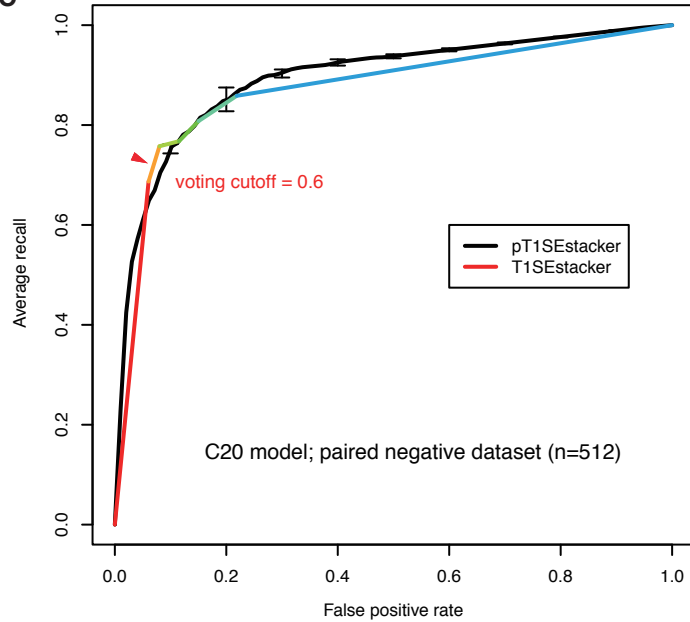

Supplement: Supplementary Figure 3 — ROC curves of T1SEstacker and pT1SEstacker models on the verified T1SEs and non-T1SEs. (A) Performance of T1SEstacker_C60 and pT1SEstacker_C60 on 99 verified T1SEs and 512 non-T1SEs. (B) Performance of T1SEstacker_C20 and pT1SEstacker_C20 on 99 verified T1SEs and paired 99 non-T1SEs. (C) Performance of T1SEstacker_C20 and pT1SEstacker_C20 on 99 verified T1SEs and 512 non-T1SEs. The best-optimized cutoff for the decision of T1SEstacker models are indicated with red arrows. [file Image_3.pdf]
